# Influence of Genipin Crosslinking on the Properties of Chitosan-Based Films

**DOI:** 10.3390/polym12051086

**Published:** 2020-05-10

**Authors:** Nataliya Kildeeva, Anatoliy Chalykh, Mariya Belokon, Tatyana Petrova, Vladimir Matveev, Evgeniya Svidchenko, Nikolay Surin, Nikita Sazhnev

**Affiliations:** 1 Department of Chemistry and Technology of Polymer Materials and Nanocomposites, The Kosygin State University of Russia, 119071 Moscow, Russia; m.solyankina@mail.ru (M.B.); nsazhnev@mail.ru (N.S.); 2 Frumkin Institute of Physical Chemistry and Electrochemistry, Russian Academy of Sciences, 119071 Moscow, Russia; chalykh@mail.ru (A.C.); petrttt@mail.ru (T.P.); matveev46@yandex.ru (V.M.); 3 Engelhardt Institute of Molecular Biology, Russian Academy of Sciences, 119071 Moscow, Russia; 4 Enikolopov Institute of Synthetic Polymeric Materials, Russian Academy of Sciences, 119071 Moscow, Russia; evgensv@yandex.ru (E.S.); niksurin@yandex.ru (N.S.)

**Keywords:** chitosan, polymeric films, crosslinking, genipin, mechanical properties, sorption isotherm, degree of crosslinking

## Abstract

Chitosan is a promising environment friendly active polymer packaging material due to its biodegradability, exceptional film forming capacity, great mechanical strength, appropriate barrier property along with intrinsic antioxidant and antimicrobial features. Bifunctional reagent was used for producing water insoluble chitosan films. Biopolymeric films crosslinked by Genipin (Gp), which is a reagent of natural origin, should have high potential in food packaging. The influence of the ratio of functional groups in the chitosan-Gp system on film absorption in the visible and ultraviolet regions of the spectrum, sorption, physical, and mechanical properties of the films has been studied. The degree of chitosan crosslinking in the films obtained from solutions containing Gp was estimated using the experimental data on film swelling and water vapor sorption isotherms. It is demonstrated that crosslinking with genipin improves swelling, water resistance, and mechanical properties of the films.

## 1. Introduction

Chitosan is one of the most promising biodegradable biopolymers, which is an amorphous-crystalline polysaccharide consisting mainly of D-glucosamine residues and it is produced via chitin processing. It is a potentially biocompatible, chemically versatile material due to the presence of amino groups and various possible M_w_. Fungi (micromycetes) and a number of enzymes are the effective destructors of chitosan [1,2,3].

Chitosan is a promising environment friendly polymer packaging material due to its biodegradability exceptional film forming capacity, great mechanical strength, appropriate barrier property along with intrinsic antioxidant and antimicrobial features [4,5]. These properties are used in the development of materials for drug delivery and tissue engineering [6,7,8]. Chitosan is approved as a bioactive additive in many countries and it has been adopted by the Food and Drug Administration for use in wound dressings [9,10].

The presence of a primary amino group makes chitosan suitable for chemical modification by covalent attachment of different functional groups or for chemical crosslinking. Because of the large amount of hydrogen bonds, chitosan degrades before melting, so it is usually recycled in solution form. Chitosan has rather low pKa value—6.4 [11], which determines its water solubility, depending on pH. Chitosan is soluble in diluted aqueous acid solutions, which, along with fiber- and film-forming ability, ensures its processing into polymeric products. Traditionally, chitosan films are obtained by casting from diluted carboxylic acid solutions. The solution is poured onto a flat surface and the solvent is allowed to evaporate.

Films and fibers obtained from chitosan solutions are water-soluble. To convert them into insoluble form, the treatment of samples with ammonia or alkaline solutions is traditionally used, which provides deprotonation of the chitosan amino groups [12]. However, as a result of such treatment the samples not only lose water solubility, but also sharply reduce the sorption capacity. In this case, bifunctional reagents are used to obtain insoluble chitosan-based films, fibers, and other materials with a high rate of water swelling. In the presence of such reagents, a three-dimensional spatial grid of chemical bonds is formed in chitosan solutions. The presence of such a grid provides the material with high and regulated sorption capacity of water vapor, controlled release of biologically active and medicinal compounds, if any are incorporated in the material [13,14].

Covalent or ionic type crosslinking reagents are used to form such grids of spatial bonds in films. Reagents of different structure and functionality are used for covalent crosslinking of chitosan: formaldehyde, toluene-2,4-diisocyanate, epichlorohydrine, and diglicidyl ether of ethylene glycol [15,16,17]. Most of the works carried out in this direction describe the technology of synthesis of polymeric materials based on chitosan modified with dialdehyde, most often with glutaraldehyde [13]. However, there are studies that indicate the toxicity of products of interaction between chitosan and aldehyde, which is an obstacle to their use [14,18], although it has been shown [19] that the use of low, but sufficient for the loss of film water solubility, amounts of glutaraldehyde does not lead to an increase in the cytotoxicity of the polysaccharide. Thus, for the production of consumable films or food packaging materials, the above-mentioned crosslinking reagents cannot be used and it is reasonable to use less toxic reagents.

**Figure 1:**
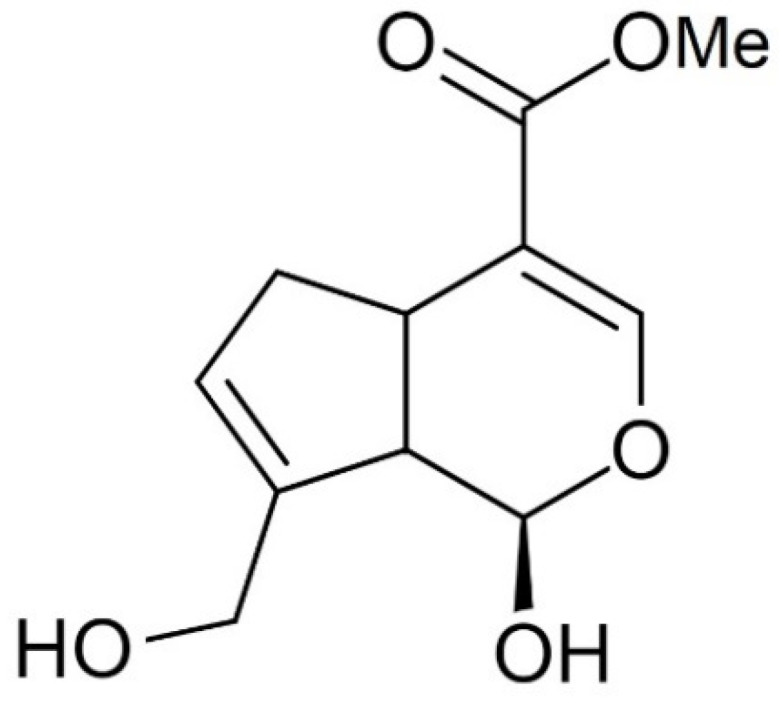
Chemical structure of genipin (Gp).

Particularly, crosslinking with genipin (Gp) (Figure 1), which is a reagent of natural origin, improves the swelling, water resistance, and mechanical properties of the films [14,20,21,22]. Genipin has recently been used in biomedical applications and for controlled drug release due to its biocompatibility and low toxicity. The low toxicity of the chitosan materials crosslinked by Gp is demonstrated by a number of studies [15,20,23,24]. Genipin crosslinked biopolymeric films should have high potential in food packaging due to these facts. However, the reversibility of the reaction of genipin with chitosan amino groups [25] and possibility of cross-linking reagent polymerization, which leads to a decrease in the number of molecules available for reaction with chitosan amino groups, require special attention to the conditions of chitosan cross-linking by genipin upon film formation.

The literature provides contradictory data regarding the impact of crosslinking on the strength and elastic properties of chitosan films. In [26], it was shown that, in comparison with non-crosslinked films derived from a mixture of chitosan/poly(ethylene oxide), a film that was based on chitosan crosslinked with genipin had greater strength and elasticity. There are reports that, when chitosan films are crosslinked with genipin, the strength increases, but the breaking elongation decreases [27,28]. In other studies on the influence of different types of crosslinking reagents on the properties of the films, including their use as food packaging [29,30,31], it is noted that, at a high degree of crosslinking, the films become more rigid, and their strength decreases. Several authors have used special techniques in order to improve the mechanical properties of the cross-linked films: introduction of nanocrystalline cellulose [32] or additional flexible-chain polymers [33]. We assume that such contradictions may be related to different initial conditions of sample formation: temperature and the solvent removal rate, the use of alkaline treatment, the amount of added crosslinking reagent, pH, etc.

In the review [31] devoted to the integrity of natural biopolymer films used in food packaging, which were obtained by crosslinking approach, the cases of different influence on the physical and mechanical properties of films of low and high cross-linking reagent concentrations are noted. Two different behaviors of soy protein cross-linked by genipin have been observed in [34]: increase in the elasticity of protein films at 1% concentration of genipin and decrease in elastic modulus at 10% of cross-linking reagent.

The aim of the present work was to study the properties of Gp cross-linked chitosan films with significantly different cross-linking reagent amounts, which might be useful in the determination of the tuning direction of biopolymer films properties for food packaging, drug delivery, or wound dressings.

The content of the cross-linking reagent affects the degree of cross-linking, the reaction rate, and time, during which the gelation process is completed in the chitosan solution. On the basis of preliminary experiments and previous studies [13], two extreme values of molar ratios of genipin-chitosan amino group (Gp/NH_2_) were chosen: the minimum, at which gelation in the used chitosan solution is possible—0.003 mol/mol, and the maximum—0.02 mol/mol, at which cross-linking is much faster, but still allows for completing the operations on preparation of the molding solution and film casting before gelation. For the films differing (almost seven times) in cross-linking reagent content, comparative studies of the form of electronic absorption spectra, sorption, structural and physical-mechanical properties of samples have been conducted. In a number of experiments, the films containing genipin in an amount exceeding the established limits, or not at all containing cross-linking reagent, were studied for a more complete interpretation of the obtained results. With the use of experimental data on film swelling and isotherms of water vapor sorption, the theoretical evaluation of the degree of cross-linking of chitosan in the films that were obtained from solutions containing Gp was carried out.

## 2. Materials and Methods

### 2.1. Materials

Сhitosan with a molecular weight of 320 kDa and deacetylation degree of 88% was purchased from Bioprogress, Voronezh Oblast, Russia. Genipin (Gp) and acetic acid ware obtained from Aldrich Chemicals, Gillingham, UK. All other reagents and solvents used were of reagent grade and they were used without further purification.

### 2.2. Experimental Method

#### 2.2.1. Preparation of Chitosan Films

The chitosan films used in the study were fabricated by means of casting/solvent evaporation technique. Chitosan was dissolved in 2 wt % aqueous acetic acid at room temperature overnight in order to obtain a 2.1 wt/v% solution. The viscous chitosan solution was filtered through filter paper to remove any undissolved gel. Chitosan solutions with a concentration of approximately 2 wt % and pH 4.1 were obtained. The solutions were kept at room temperature for 3 h for degassing. The degassed solutions were then cast onto glass plates and dried to a constant weight at ambient temperature. The thickness of the dried films was 50 ± 5 μm. The resulting films were water-soluble.

#### 2.2.2. Preparation of Genipin-Crosslinked Films (Сhitosan-Gp)

Chitosan solution was prepared, as described in Section 2.2.1. The concentration of chitosan solution was specified after solution filtration and weighing of the paper filter mass dried up until constant. Chitosan solution containing 0.2 g of polymer was mixed with 1.25 mL of aqueous solution of genipin obtained by dilution of its 0.5% aqueous solution. Table 1 shows concentrations of genipin solution. The obtained solutions were kept at room temperature for 30 min, and then poured onto a glass disk and left for crosslinking and solvent evaporation. The resulting films were dried at an ambient temperature to a constant weight (after 3 weighing every 2–3 h). The films considered ready were obtained after 70 ± 5 h. The thickness of the films was determined while using the EP-10–60 Ms thickness indicator. The scale interval was 0.01 mm. The thickness of the dried films was 52 ± 5 μm.

#### 2.2.3. Spectrophotometric Studies

The absorption spectra of the pure chitosan and genipin crosslinked chitosan (chitosan-Gp) films were recorded on a Shimadzu UV-2501PC spectrophotometer (Kyoto, Japan) in the spectral region from 190 to 800 nm.

#### 2.2.4. Swelling Studies

The swelling kinetics of chitosan and genipin crosslinked chitosan films with the size of 2 × 2 cm^2^ in water were gravimetrically studied. Measurements were made on HR-200 A&D Co. LTD scales (Tokyo, Japan). Before the sorption experiments, the films were vacuumized to a constant weight at a residual pressure of ~10^−2^ Pa and temperature of 120 °C. Preliminary thermogravimetry measurements showed that the residual water content in chitosan and genipin crosslinked chitosan films before the experiments did not exceed 0.2 wt %. All of the samples were conditioned in a dry desiccator at zero humidity before measurements. The swelling ratio of each studied chitosan membrane was determined by immersing the membrane in water (pH = 7.0) at room temperature. The swelling ratio of the chitosan membrane was calculated, as follows:

(1) $$M\left(\mathrm{wt}\;\%\right)=\frac{m-m_{0}}{m_{0}}\times 100$$

where *M* (%) is the percentage water absorption of the film; and, *m*_0_ and *m* are the weights of the samples in the dry and swollen states, respectively. Each swelling experiment was repeated three times, and the average values are reported. A cross plot of swelling degree on time *M* = f(t) was constructed according to the obtained data.

#### 2.2.5. Sorption Measurements

McBen vacuum scale was used to study the processes of interaction of water vapor with chitosan and genipin crosslinked chitosan membranes (2 × 5 cm^2^). The quantity of sorbed (desorbed) water vapors was determined by stretching of the calibrated quartz spiral with the help of optical registration system. The accuracy of measurement was ±0.00001 g. The sorption measurements were performed under isobaric-isothermal conditions in desiccators at a temperature of 20 ± 1 °C. The methods of integral and interval sorption were used [35]. All of the samples were conditioned in a dry desiccator at zero humidity before taking measurements. The range of relative changes in water vapor pressure (p/ps) was changed from 0.20 to 0.95. The sorption of the chitosan membrane was calculated as (1).

#### 2.2.6. Mechanical Property Measurements

Rectangular specimens of 5 × 1 cm^2^ of chitosan and genipin crosslinked chitosan membranes were used to determine the limiting strength characteristics. Single axis tensile testing at a deformation rate of 10 mm/s was carried out on a universal testing machine BM-50 (Russia) with the fixation of tensile strength and elongation at break. The experiment was carried out five times (tensile strength at break point was determined on Russian state standard (GOST) 11262-80 type 5 blades).

#### 2.2.7. Structural-Morphological Studies

Structural-morphological studies were carried out by the method of transmission electron microscopy. The samples were etched in oxygen discharge plasma for 40 min. to reveal the fine structure (etch depth of 150–200 nm). Electron energy in the etching zone was 5–6 eV, oxygen pressure was 0.1 mm Hg, etching time was 20 min. Imaging of the etched surface was carried out by transmission electron microscopy while using single-stage carbon-platinum replicas using the EM 301 (Philips, Dutch, The Netherlands) electron microscope.

## 3. Results and Discussion

### 3.1. Optical Properties of the Gp Crosslinked Chitosan Films

**Figure 2:**
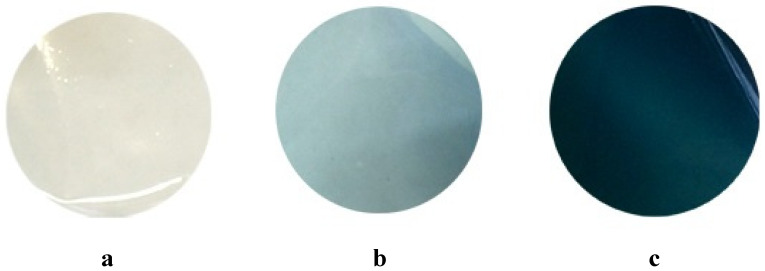
Photographs of the studied films. Pure chitosan film (**a**); chtosan films, crosslinked in Gp/NH_2_ molar ratio of 0.003 (**b**) and 0.02 (**c**).

In contrast to practically colorless films of pure chitosan, the films with introduced Gp are characterized by pronounced blue color, the intensity of which depends on the Gp/NH_2_ ratio (Figure 2). Such coloration is typical for various products of genipin and chitosan interaction: hydrogels, microparticles, etc. [20].

**Figure 3:**
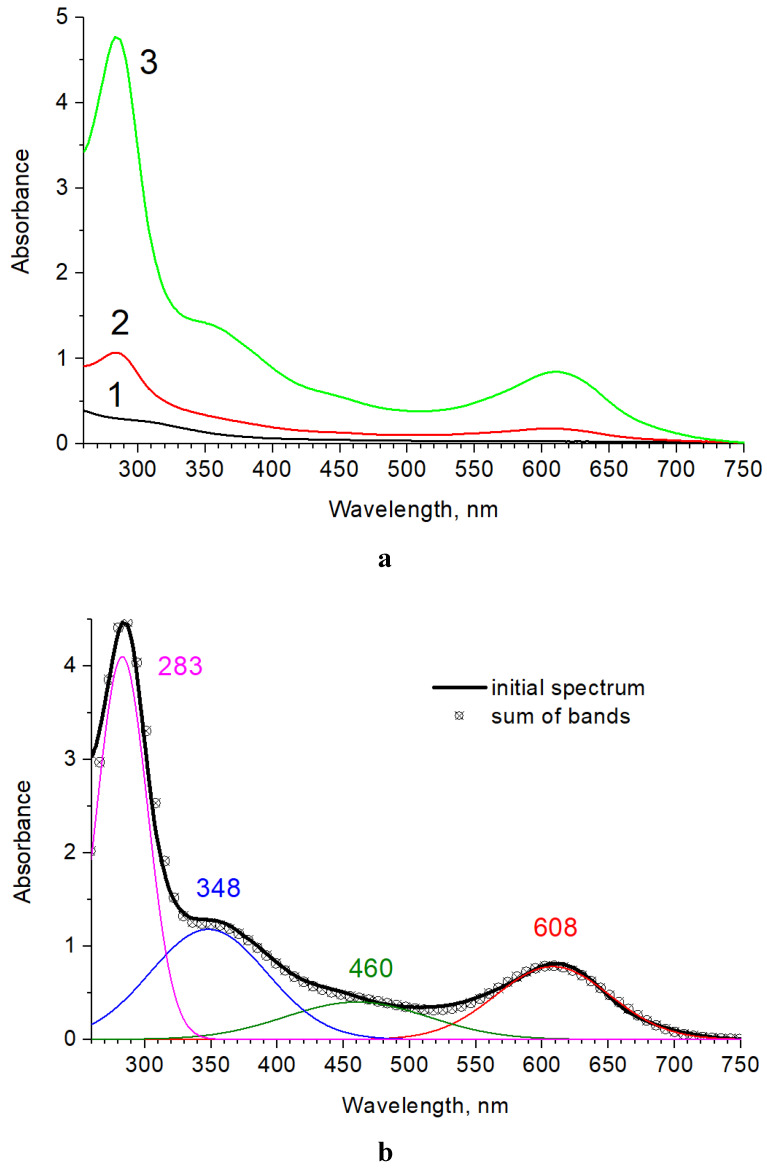
(**a**) UV-vis absorbance of chitosan film (1), and the films obtained at Gp/NH_2_ molar ratios of 0.003 (2) and 0.02 (3). (**b**) Deconvolution of the absorbance spectrum for the film with Gp/NH_2_ molar ratio of 0.02.

Figure 3a shows the electronic absorption spectra of films that are based on chitosan crosslinked with genipin at different Gp/NH_2_ ratios. The absorption spectrum of pure chitosan exhibits weak bands in the area below 400 nm. Intensive absorption bands in the range of 260–700 nm appear in films with Gp (Figure 3a).

For the analysis, the pure chitosan absorbance spectrum was subtracted from the spectra of crosslinked films. The obtained spectra are well approximated by a sum of four Gaussian bands: A_608 nm_ = 0.78; A_460 nm_ = 0.4; A_348 nm_= 1.18; and, A_283 nm_= 4.1, which is illustrated in Figure 3b for the film with the Gp/NH_2_ ratio of 0.02.

The form of the film absorption spectrum with a Gp/NH_2_ ratio of 0.003 is similar to the one that is discussed above, which testifies to the identical set of chromophore groups in the samples under consideration and indicates the same nature of chemical reactions occurring during crosslinking with different genipin/chitosan ratio. The ratio of absorption bands intensity in the studied films is close to the ratio of genipin concentration in them and is about 6.

A similar form of the absorption spectra was previously observed upon crosslinking performed in solutions [36]. In [21], the absorption band at 280–290 nm was associated with the inclusion of chitosan amino group in the heterocycle of genipin, which leads to the formation of heterocyclic amine, and the absorption band at 610 nm was assigned to the reaction of radical polymerization of genipin, induced by oxygen in air, occurring as one of the stages in the process of the crosslinking of chitosan. The last absorption band determines the intense blue color of the genipin crosslinked chitosan films. The increase of the peak intensity at 280–290 nm indicates an increase in the degree of binding of Gp by chitosan at a high Gp/NH_2_ ratio.

### 3.2. Kinetics of the Swelling and Water Vapor Sorption by the Films

**Figure 4:**
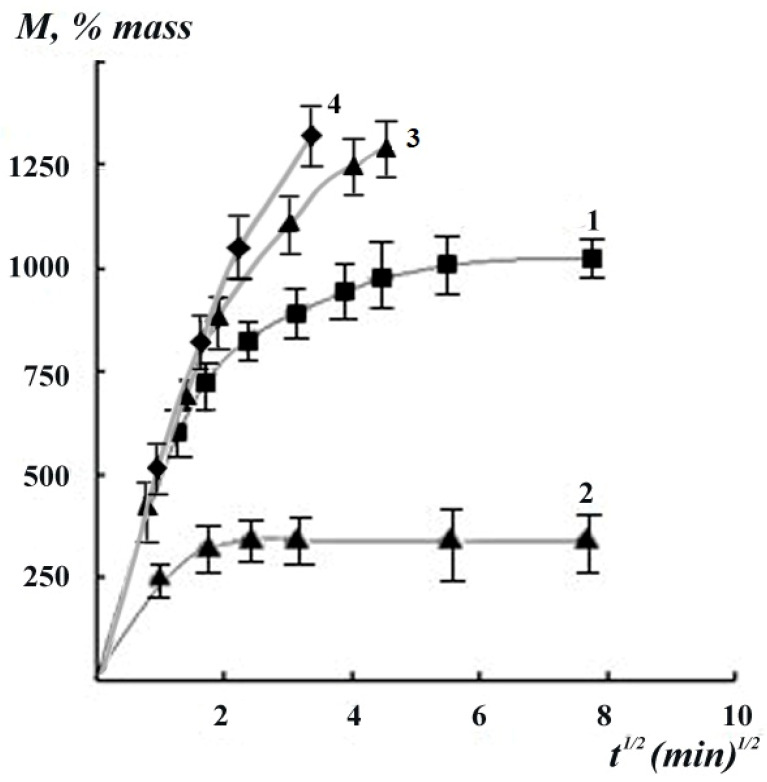
The kinetics of swelling of genepin crosslinked chitosan films with Gp/NH_2_molar ratios of 0.003 (1), 0.02 (2), 0.002 (3), and chitosan film without genipin (4) in water.

The nature of the swelling kinetic curves of Gp cross-linked chitosan films in water (Figure 4) shows that films containing Gp at molar ratios of 0.003 and 0.02 mol/mol exhibit limited swelling in water, and the degree of their swelling at the initial stage of the process is linearly dependent on t^1/2^, which indicates a Fick diffusion mechanism of water absorption. The equilibrium swelling decreases with an increasing concentration of cross-linking reagent: 1020% wt. for film with Gp/NH_2_ ratio of 0.003 and 340% wt. for Gp/NH_2_ of 0.02 mol/mol. This clearly indicates an increase in grid density in cross-linked Gp films. Upon a decrease of the genipin content to 0.002 mole/mole, the film dissolves in water, its kinetic curve is similar to that of an unrestricted swelling of a non-crosslinked film (curves 3 and 4, Figure 4).

**Figure 5:**
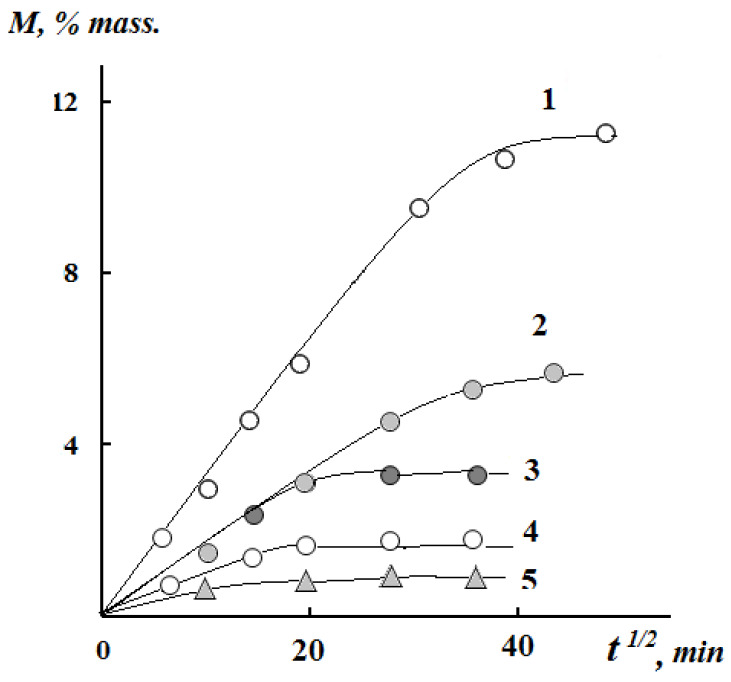
Kinetics of water vapor sorption of genipin crosslinked chitosan films crosslinked: 1—Gp/NH_2_ ratio 0.003 mol/mol; 2—0.02 mol/mol. 1, 4—Gp/NH_2_ ratio of 0.02 mole/mol; 2,3,5—0.003 mole/mole at 293 K and P/P_0_ 0.2(5), 0.3(4), 0.6(3), 0.7(1), 0.8(2).

Figure 5 shows the kinetic curves of interval sorption of water vapor for chitosan films crosslinked with genipin at different ambient humidity. It can also be seen that the Fick kinetics of the sorption equilibrium establishment is observed for all types of crosslinked and non-crosslinked samples in these conditions. The difference in sorption kinetics and swelling kinetics is only in the speed of sorption equilibrium establishment. It is most vividly manifested in the values of translational coefficients of water diffusion, which we estimated from experimental data M–t^1/2^, while using Equation (2)

D = 1.96·L^2^/π^2^t_0.5_ (2)

where L is the membrane thickness and t_0.5_ is the time it takes to reach half saturation of the membrane sorption capacity.

**Figure 6:**
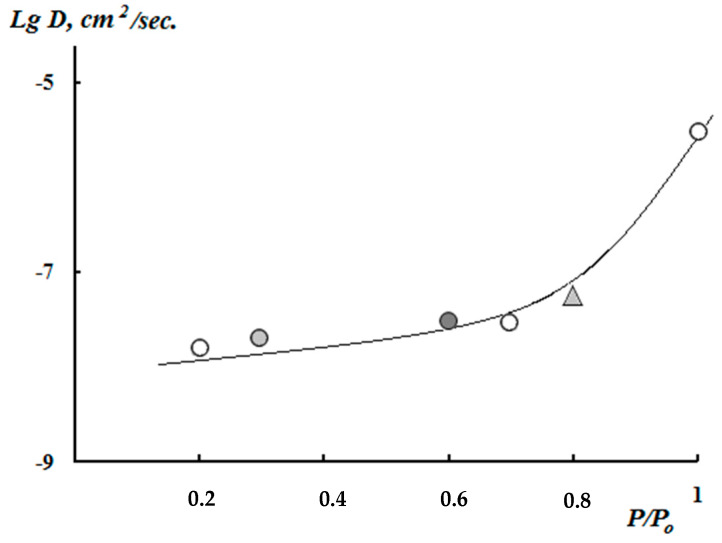
Dependence of the water molecule diffusion coefficients in the genipin crosslinked chitosan films on the ambient humidity.

Figure 6 shows the coefficients of water diffusion in chitosan films, depending on the relative humidity of the environment. It is seen that, with increasing humidity, the translational mobility of sorbed water molecules increases, which indicates the plasticization of chitosan films crosslinked with genipin. Obviously, this result indicates that it is possible to apply the Flory–Riener Equation (3) to calculate the chemical bonds grid density of chitosan films crosslinked with genipin.

**Figure 7:**
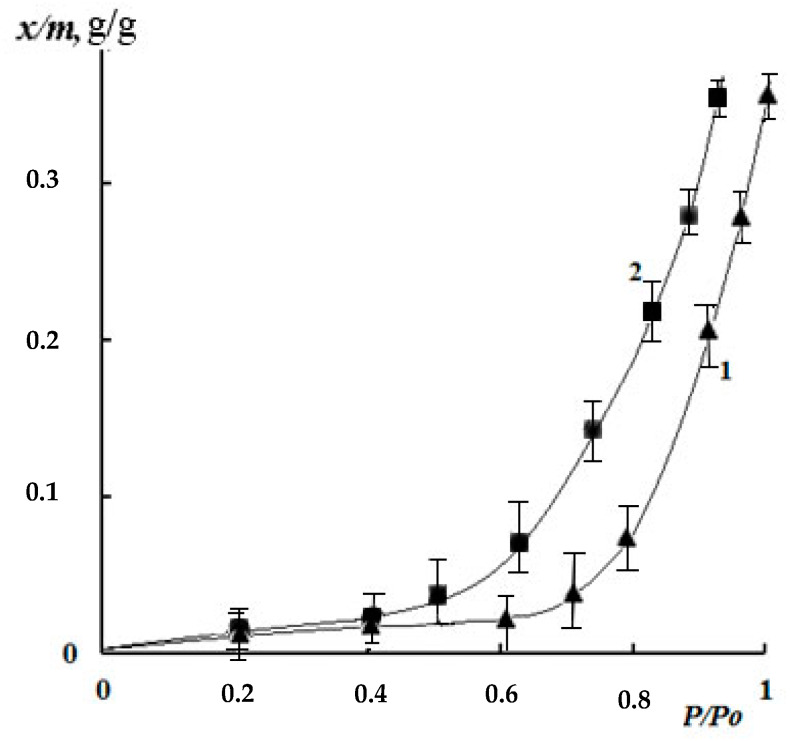
Isotherms of water vapor sorption of chitosan films, crosslinked by genipin. 1—Gp/NH_2_ ratio 0.02 mol/mol; 2—0.003 mol/mol.

**Figure 8:**
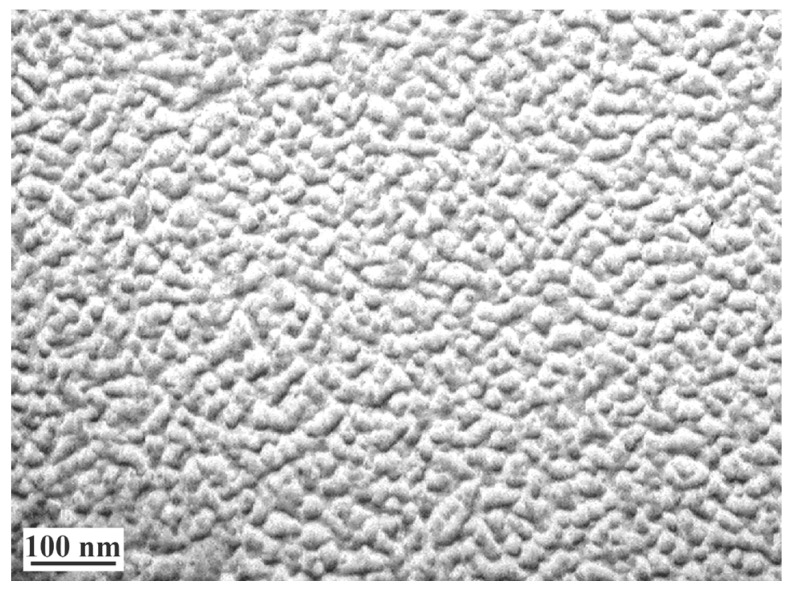
Microphotograph of the supramolecular structure of the same formed with Gp/NH_2_ molar ratio of 0.003.

Figure 7 shows the isotherms of water vapor sorption by the films obtained from 2% solutions of chitosan with different content of cross-linking reagent: Gp/NH_2_ of 0.003 and 0.02 mol/mol. It can be seen that, for all samples of genipin crosslinked chitosan films, the isotherms of water vapor sorption are *S*-shaped. In this case, their sorption capacity decreases with increasing amount of genipin in the whole interval of water vapor pressure. Since, genipin crosslinked chitosan films are non-porous sorbents with homogeneous nanoscale domain structure (Figure 8), according to electron microscopic studies, we did not consider it expedient to use the BET model to analyze the sorption isotherms.

**Figure 9:**
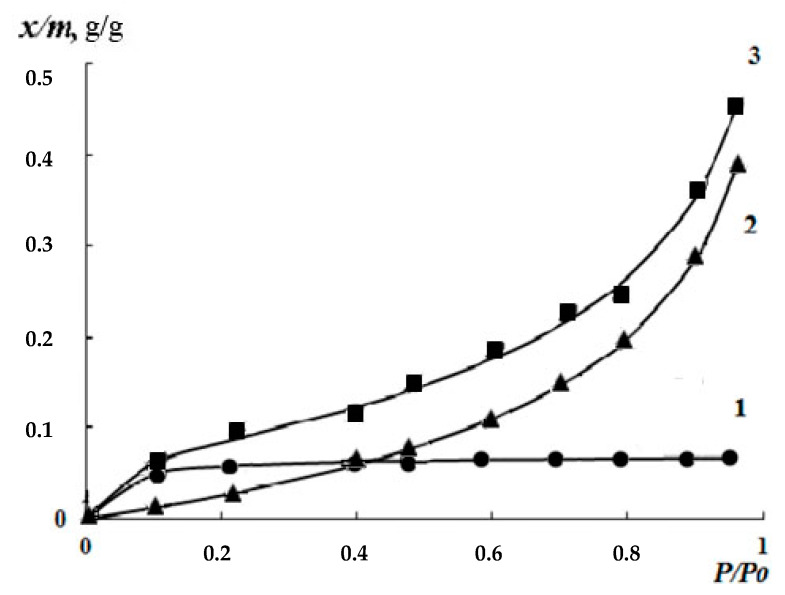
Decomposition of the film water vapor sorption isotherm (3) in the framework of the Flory–Haggins model: 1—Langmuir isotherm, 2—Flory-Haggins isotherm.

Following the model of double sorption and the method proposed earlier [35], decomposition of the sorption isotherms in the TWOFL package into Langmuir and Flory–Haggins components was performed (Figure 9). It can be seen that the filling of accessible (free) functional groups of chitosan with water molecules is realized in the region of low vapor activity, whereas, in the region of high humidity, the main fraction is made up of free and mobile mode molecules. The numerical values of paired interaction parameters χ obtained for the Flory–Haggins mode amounted to 0.85 for Gp cross-linked chitosan at 0.003 mol/mol and 0.99 for 0.02 mol/mol ratios. It is important to note that the critical value for aqueous solutions of chitosan amounts to 0.5 [35]. It can be assumed that this difference in the numerical values of the paired interaction parameter is due to the contribution of genipin molecules to the intermolecular interactions of the system components.

We calculated the molecular mass of the chemical bond grid between the nodes M_c_ using the values of the equilibrium degree of swelling (Figure 4) and the paired interaction parameter χ according to Flory–Riener Equation (3) [37].

(3) $$\ln(1-\phi_{2})+\phi_{2}+\chi_{1}\phi_{2}^{2}+\frac{d_{2}{\bar{V}}_{1}}{M_{c}}\left(\phi_{2}^{1/3}-\frac{2\phi_{2}}{f}\right)=0$$

where *φ*_2_ is the volume fraction of the polymer in the swollen sample; *d*_2_ is the polymer density; ${\bar{V}}_{1}$ is partial molar volume of the solvent; *M_c_*—molecular weight of a chain segment between nodes of the grid; and, $\chi_{1}$; *f* is the grid functionality. The density of the chitosan films (~1.021 g/cm^3^), crosslinked with genipin (~1.018 g/cm^3^) were determined by hydrostatic weighing; the partial mole volume of sorbate was taken equal to mole volume of water in the calculations. The volume fraction of the polymer was calculated within the limits of additive model of density of swollen sorbent. Table 2 provides the obtained results.

The values of the molecular weight of the grid between the mesh nodes M_c_ can be calculated while using the values of the equilibrium degree of swelling (Figure 4) and the paired interaction parameter χ obtained from sorption isotherms according to the Flory–Riener equation [37].

The change in the degree of modification of amino groups is proportional to the changes in the content of crosslinking reagent in chitosan solution; thus, the molar ratio Gp/NH_2_ can be used to assess the degree of crosslinking, as can be seen from Table 2. However, the absolute values of the number of meshes 2.7 and 18.8 exceed the maximum possible degree of crosslinking of chitosan macromolecules, which amounts to 6 and 42 in calculation per 100 elementary links for films obtained while using different Gp/NH_2_ molar ratios. This indicates that intermolecular contacts, more precisely hydrogen bonds, make an additional contribution to the formation of the grid of meshes. Chemical crosslinking only fixes the structure of the film formed by the rapprochement of the macromolecules due to evaporation of the solvent.

The non-equilibrium domain structure of the polymer would be fixed if the interaction with Gp that leads to the chemical crosslinking of macromolecules occurs before the equilibrium arrangement of the macromolecular chains (Figure 8). If the crosslinking is completed with low residual solvent content, when the mesh grid is already mostly formed, the more perfect equilibrium structure of the film is fixed, and one can expect that such material will have a higher level of physical and mechanical properties. This fact is important for the technology of obtaining polymer materials from solutions while using crosslinking reagents and it provides a tool for programming the properties of polymer materials.

### 3.3. Mechanical Properties of Gp-Crosslinked Chitosan Films

To obtain water-insoluble films, except for the Gp/NH_2_ ratio of 0.02, the minimum Gp/NH_2_ molar ratio of 0.003, which provides crosslinking of chitosan in the solution, and a ratio of 0.0025, the use of which did not lead to gelation in the solution. The concentration of a solution increases upon film formation in the process of the solvent evaporation, and smaller quantity of cross-linking reagent, than for gel formation in 2–5% solutions of chitosan, is sufficient for the completion of cross-linking reaction. After the evaporation of the solvent, the film obtained at this Gp/NH_2_ ratio remained colorless during 24 h, but it later acquired blue coloring and water resistance. The films, obtained at lower genipin content (0.002 mol/mol ratio), are water soluble (Figure 4).

Obviously, the crosslinking of chitosan by bifunctional reagent in solution fixes mutual arrangement of polymer chains, not allowing them to take equilibrium conformations in the process of solvent evaporation. This fact is confirmed by the revealed sharp decrease in the level of physical and mechanical properties of films: the strength of films obtained using Gp/NH_2_ molar ratios of 0.003 and 0.02 is much lower than the strength of films that were obtained in conditions of polymer chains relaxation in the absence of crosslinking reagent and slow evaporation of solvent at 20 °C. However, reducing the content of genipin in chitosan solution to the concentration at which the evaporation of the solvent occurs faster than the completion of crosslinking of macromolecular chains of chitosan not only does not lead to a decrease in strength, but even increases the strength of the film, probably due to the fixation of macromolecular chains in an equilibrium position, in which the intermolecular interactions are realized to a higher degree (p. 4, Table 3). The ratio of 0.0025 makes it possible, on the one hand, to obtain a film insoluble in water and, on the other hand, to increase the breaking load under certain temperature (20–22 °C) and humidity conditions. Similar data were obtained in [34] in the study of soy protein films that were obtained in the presence of genipin: the improvement of mechanical properties only occurred when the content of the cross-linking reagent was less than 1%. With changing conditions of chitosan Gp crosslinking reaction, the optimal ratio of functional groups can be different.

## 4. Conclusions

The influence of the ratio of functional groups in the chitosan-cross-linking reagent Gp system on the film absorption in the visible and ultraviolet spectral regions, sorption, physical, and mechanical properties of the films was studied at significantly different concentrations of cross-linking reagent 0.02 and ≤ 0.003 mol/mol ratios. The analysis of electronic absorption spectra showed the formation of an identical set of chromophore groups in the studied samples, which indicates the same nature of chemical reactions occurring during cross-linking in systems, with a genipin/chitosan molar ratio that varies by a factor of 6.7. Kinetic and thermodynamic parameters of swelling and water vapor sorption processes (diffusion coefficients, equilibrium sorption values, and paired interaction parameters χ) change in proportion to the cross-linking reagent content. The decomposition of sorption isotherms into Langmuir and Flory–Haggins components within the double sorption model according to the proposed technique has shown that the filling of chitosan functional groups with water molecules is realized in the region of low vapour activity, while the main fraction is made up of molecules of free and mobile mode in the region of high humidity. At the same time, genipin cross-linking contributes to the intermolecular interactions, as evidenced by the difference in numerical values of the paired interaction parameter χ for different genipin contents in the films (0.85 at Gp/NH_2_ ratio of 0.003 mol/mol, and 0.99 for 0.02 mol/mol). The cross-linking of the chitosan by a bifunctional reagent in the solution fixes the mutual location of polymer chains, not allowing them to take equilibrium conformations during solvent evaporation, which leads to a deterioration in the physical and mechanical properties of the resulting films. Only at a very low content of the cross-linking reagent (0.0025 mol/mol), at which there is no gel formation in the molding solution, the fixation of macromolecular chains occurs in the position where intermolecular interactions are realized to the greatest extent, which, in its turn, leads to a significant increase in the strength of chitosan films. The results of research into the properties of chitosan films with significantly different cross-linking reagent contents can be used to determine the direction of programming of cross-linked biopolymer film properties for food packaging, edible coatings, or for medical use to increase strength and regulate moisture absorption.

## Figures and Tables

**Table 1:** Concentrations of genipin solution used for genipin cross-linked chitosan film production.

Ratio Gp/NH_2_, mol/mole	Genipin Solution Concentration, %	Genipin Content of the Molding Solution, % of Chitosan Mass
0.02	0.24	1.5
0.003	0.036	0.225
0.0025	0.03	0.187
0.002	0.024	0.15

**Table 2:** The results of the degree of chitosan crosslinking by genipin calculation.

Molar Ratio Gp/NH_2_	Equilibrium Swelling Degree, %	Paired Interaction Parameter χ	Molecular Weight of the Chitosan Chains between the Mesh Nodes М_с_, g/mole	Number of Elementary Links of Chitosan between Mesh Nodes n_c_	Number of Meshes per 100 Elementary Links of Chitosan
0.003	1025	0.85	6934	42	2.7
0.02	340	0.99	1015	6	18.8

**Table 3:** Mechanical properties of the chitosan films formed at different Gp/NH_2_ ratios.

Gp/NH_2_ Molar Ratio	Breaking Load, MPa	Breaking Elongation, %
0	73	33
0.02	65	33
0.0030	55	25
0.0025	90	15
